# Synthesis of long group IV semiconductor nanowires by molecular beam epitaxy

**DOI:** 10.1186/1556-276X-6-113

**Published:** 2011-02-02

**Authors:** Tao Xu, Julien Sulerzycki, Jean Philippe Nys, Gilles Patriarche, Bruno Grandidier, Didier Stiévenard

**Affiliations:** 1 Département ISEN, Institut d'Electronique, de Microélectronique et de Nanotechnologie, IEMN (CNRS, UMR 8520), 41 bd Vauban, 59046 Lille Cedex, France; 2 CNRS-Laboratoire de Photonique et de Nanostructures, Route de Nozay, 91460 Marcoussis, France

## Abstract

We report the growth of Si and Ge nanowires (NWs) on a Si(111) surface by molecular beam epitaxy. While Si NWs grow perpendicular to the surface, two types of growth axes are found for the Ge NWs. Structural studies of both types of NWs performed with electron microscopies reveal a marked difference between the roughnesses of their respective sidewalls. As the investigation of their length dependence on their diameter indicates that the growth of the NWs predominantly proceeds through the diffusion of adatoms from the substrate up along the sidewalls, difference in the sidewall roughness qualitatively explains the length variation measured between both types of NWs. The formation of atomically flat {111} sidewalls on the <110>-oriented Ge NWs accounts for a larger diffusion length.

## Introduction

Semiconductor nanowires (NWs) consist of a solid rod with a diameter usually smaller than 100 nm and a length that can vary from the nanometer to the millimeter-scale depending on the technique used to synthesize the rods. Although, for the majority of the NWs, their growth is described by the vapor-liquid-solid mechanism, based on the catalytic effect of a metal seed particle, their length is mostly related to the way the chemical compounds are supplied. In chemical vapor deposition (CVD), a wide range of partial pressures for the reactive source gases can be used. As a result, Si NWs with a millimeter-scale length have been successfully synthesized with a reasonable time [1]. Conversely, when elemental compounds are supplied instead of gas precursors, as it is the case for the growth of NWs in molecular beam epitaxy (MBE), ultra high vacuum (UHV) conditions are required. The pressure in the system is around 10^-9 ^times smaller than in a CVD chamber and the NW length typically does not exceed a few micrometers [2,3].

**Figure 1:**
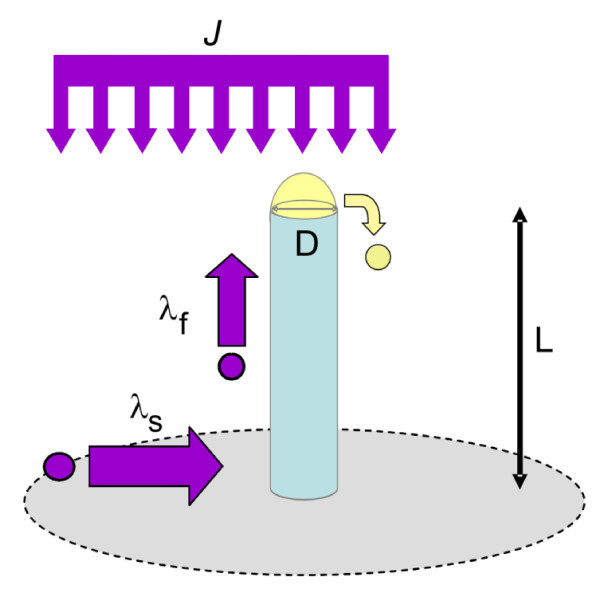
**Model of group IV semiconductor NW growth by MBE**. The NW has a length *L *and a diameter *D*. Elemental compounds are evaporated with a rate *J *and impinge on the surface as well as on the Au seed particle, positioned at the top of the NW. Once adsorbed, the adatoms diffuse on the substrate and on the NW sidewalls with diffusion lengths λ_s _and λ_f_, respectively. Au adatoms can also diffuse away from the seed particle.

As illustrated in Figure 1 for the MBE growth, the ratio between the exposed surface of a seed particle and the collection area between the seed particles is usually small. Due to the low pressure in the growth chamber, the direct impingement of elemental compounds onto the seed particle has a small probability to occur. Therefore, growth predominantly proceeds from the diffusion of elemental compounds that adsorb on the substrate between the seed particles. The adatoms reach the seed particles after diffusing on the substrate and the sidewalls with different diffusion length coefficients, λ_S _and λ_f_, respectively. As surface diffusion is a rather slow process, it is only because the crystallization at the interface between the seed particles and the NW is high enough that NWs emerge from the film growth. Such mass transport mechanism yields a NW length that is inversely proportional to the NW diameter [3,4].

In contrast to MBE grown III-V semiconductor NWs that can reach micrometer-scale lengths, group IV semiconductor NWs, which are generally grown with a <111> orientation, show a much smaller length [4,5], making their integration into devices more difficult. In a purpose to understand the physical mechanisms that prevent the fabrication of group IV semiconductor NWs with micrometer-scale lengths, we investigated the growth of Si and Ge NWs on a Si(111) surface with the MBE technique. In this article, we show that not only <111> but also <110>-oriented Ge NWs are grown on the Si(111) surface. Surprisingly, the length of the latter can reach a few micrometers. From a comparative study of differences in the structural morphology between <111> and <110>-oriented NWs, we are able to explain why <110>-oriented NWs grow longer.

## Experimental details

The SiNWs were fabricated by the MBE method using gold droplets. The gold droplets were formed directly by gold deposition on a heated Si(111) surface in UHV at a gold deposition pressure of 6 × 10^-10 ^mbar. The density and the diameter of the gold droplets are determined by the Au evaporation rate and the temperature of the samples via the Ostwald ripening process [6]. In the next step, the growth of the NWs was achieved from the sublimation of Si or Ge at a deposition pressure of 10^-9 ^mbar. In order to grow the NWs, the Si(111) surface was heated either at 550°C for the growth of Si NWs or at 350°C to obtain Ge NWs. The evaporation rate of elemental Si and Ge was measured from the thickness of the two-dimensional film grown on the substrate during the NW growth.

The morphology of the NWs was investigated by electron microscopies: scanning electron microscope (SEM) and high-resolution transmission electron microscope (HRTEM). To perform the HRTEM experiments, the NWs were cleaved with micromanipulators in the chamber equipped with a focus ion beam machine and transfer into holey grids covered with a very thin carbon layer.

## Results and discussion

**Figure 2:**
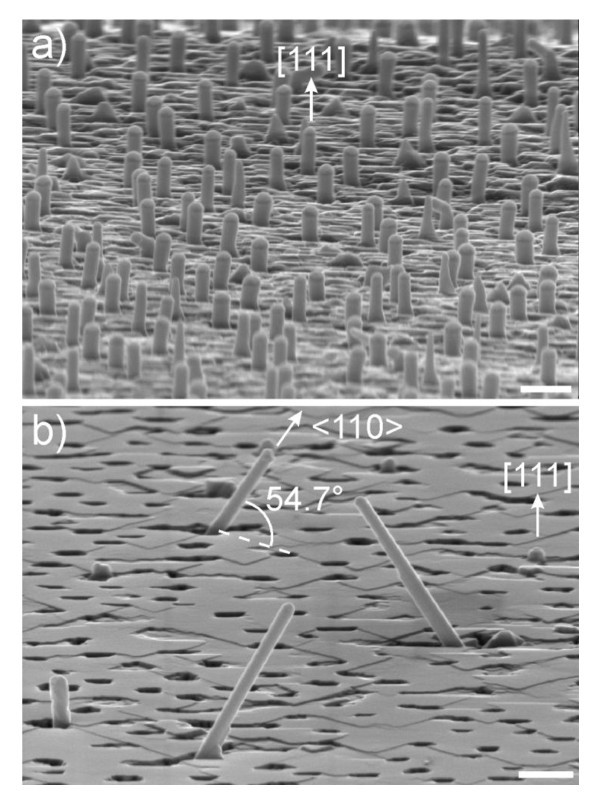
**SEM images of (a) Si NWs and (b) Ge NWs grown on a Si(111) surface by MBE**. The orientations of the NWs are indicated in the SEM images. The growth times were 2 and 1 h for the Si and Ge NWs, respectively. The scale bars correspond to 400 nm.

**Figure 3:**
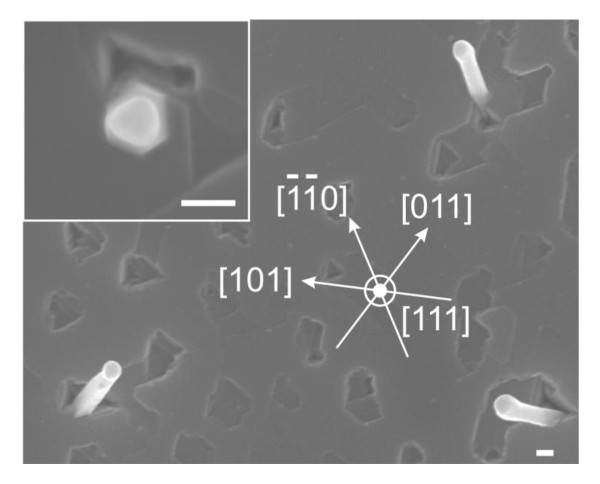
**Top view SEM images of Ge NWs grown on a Si(111) that show the different growth direction**. The scale bars correspond to 100 nm.

Tilted views of the post-growth Si(111) surfaces are shown in Figure 2. When Si is sublimated, the majority of the Si NWS are found to be perpendicular to the Si(111) surface (Figure 2a). Their growth axis is thus along the [111] direction, in agreement with previous observations [4,5]. In contrast, the growth of Ge NWs leads to two different kinds of growth directions (Figure 2b). The shortest NWs usually show the Au seed particle just above the overgrown Ge film. These NWs appear normal to the surface when observed in top view SEM images such as the one shown in the inset of Figure 3. They thus grow along the [111] direction, likewise the Si NWs. As for the second type of Ge NWs, these NWs are much longer and point at 54.7° from the surface plane. In the top view SEM image of Figure 3, these inclined Ge NWs are found to grow along three different directions only. When projected in the (111) surface plane, the directions make an angle of 60°. Therefore, these Ge NWs are oriented along one of the equivalent <110> directions, in agreement with the growth of <110>-oriented Ge NWs obtained on the Ge(111) surface by MBE [7].

Group IV semiconductors NWs have been found to grow with different orientations, depending on the temperature of the surface during the growth and the evaporation rate of elemental Si or Ge. While a high surface temperature favors the growth of <111>-oriented NWs, contradictory results have been obtained regarding the effect of the evaporation rate [7,8]. However, consistent with these previous studies, we note that the comparison of the length between the <111>-oriented Ge NW and those oriented along the <110> directions shows a strong difference. In our study, the first types of NWs are rarely higher than the overgrown Ge layer, while for the second type of NWs, the part of the NWs that surpasses the overgrown Ge layer can reach a length of 2 μm when the growth time is 60 min and the deposition rate is about 1.7 Å/s. The growth direction of the NWs thus seems to play an important role on the maximum length that group IV semiconductor NWs can reach. Although the deposition rate of Si was 0.5 Å/s in Figure 2a, a two-hour growth does not allow to build NWs higher than 400 nm, consistent with previous results that reported the difficulties to grow <111>-oriented Si NWs longer than 500 nm by MBE [9].

**Figure 4:**
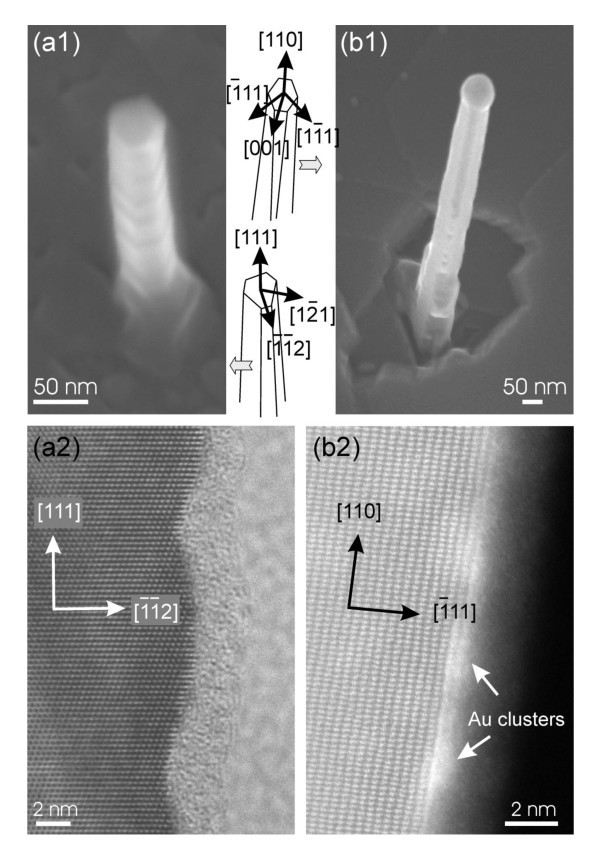
**SEM observation of NW sidewalls for (a1) a <111>-oriented Si NWs and (b1) a <110>-oriented Ge NWs grown on a Si(111) surface by MBE**. Some sidewall orientations are indicated for both types of NWs. Lattice-resolved TEM images showing the roughness of **(a2) **a {112} sidewall on a <111>-oriented Si NWs and **(b2) **a {111} sidewall on a <110>-oriented Ge NWs.

In order to understand the physical origin of the growth direction dependence on the NW length, we examined the sidewalls of both types of NWs, as shown in Figure 4. The <111>-oriented Si NWs consist of six sidewalls that exhibit small facets. It has been shown that these sidewalls correspond to {112} planes [9]. For <111>-oriented Si NWs grown by CVD at low silane partial pressure, that show similar sidewall orientations [10], gold is known to diffuse from the seed particle and to wet the sidewalls [11,12]. Adsorption of gold on Si(112) planes is also known to causes the faceting of these planes [13]. Similarly, Au diffusion from the seed particle is at the origin of the facet formation on the {112} planes, for which the crystallographic orientations alternate between {111} planes and high index planes [14]. HRTEM images of the sidewalls for the MBE grown Si NWs are consistent with the observations performed on <111>-oriented Si NWs grown by CVD. For example, Figure 4a2 reveals the rough morphology of one of the {112} sidewalls. Although the facets are rather rounded, probably due to the oxide layer that covered the sidewall, a corrugation of up to 2 nm is found when the height profile of the sidewall is measured.

As for the <110>-oriented Ge NWs, they also exhibit an irregular hexagonal cross-section, but the orientations of the sidewalls are different. They consist of {111} and {100} planes, where two {100} planes are opposite to each other and are separated on each side by two adjacent {111} planes [15,16]. In addition, the {100} planes are usually narrower than the {111} planes and their width decreases toward the base of the NWs, which undergoes the longest exposure time to the Ge deposition and diffusion (Figure 4b1). As the {111} planes are the dominant sidewalls, these sidewalls were investigated by HRTEM. Figure 4b2 shows that the {111} sidewalls are atomically flat. Scattered bright clusters are also seen superimposed to the atomic lattice and indicate the presence of Au-rich clusters.

Similarly to the <111>-oriented Si NWs, gold diffuses from the seed particle to wet the sidewalls of the <110>-oriented Ge NWs. However, in contrast to the adsorption of gold on the {112} planes, the adsorption of gold on the Si and Ge (111) surfaces does not produce a roughening of the surface. Instead, atomically flat (111) surfaces are generally observed with a √3 × √3 reconstruction, when the Au coverage is higher than one monolayer [17-19]. Our observation of a flat sidewall is thus consistent with previous surface studies about the adsorption of gold on group IV semiconductor (111) surfaces. In addition, as the {111} sidewalls contain Au-rich clusters, we might expect to have more than one monolayer of gold adsorbed on the NW sidewalls. Such result suggests that the formation of a √3 × √3 reconstruction between the clusters occurs during the NW growth in UHV.

**Figure 5:**
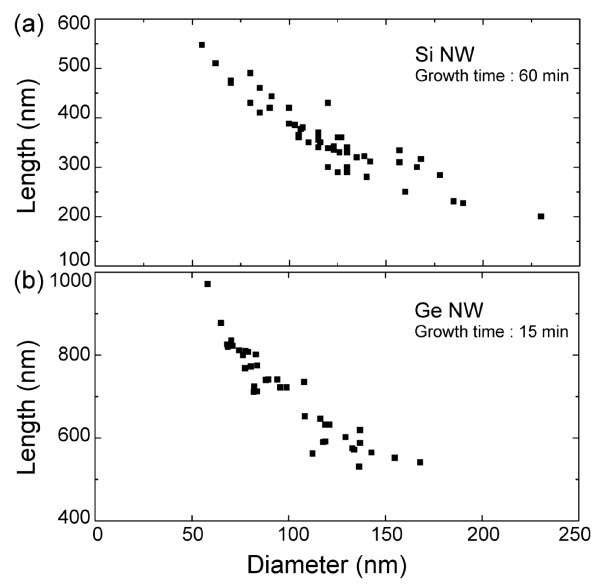
**Correlation between the length and the diameter of <111>-oriented Si NWs and <110>-oriented Ge NWs**. For Ge NWs, the data are measured for two different growth times. The evaporation rate for both experiments was 1.5 ± 0.2 Å/s.

As already described in the introduction, the growth of NWs by MBE predominantly proceeds through the diffusion of adatoms that adsorb on the substrate in between the NWs. Indeed, if we consider the surface of the seed particle exposed to the flux of elemental compounds and the area surrounding the NWs that serves as a reservoir to collect adatoms for the NW growth, their ratio is usually quite small. When the NW is still short, typically at the beginning of the growth, the contribution of the adatoms diffusing from the substrate up along the NW is thus the strongest to the growth rate. This mechanism implies that the crystallization rate at the interface between the seed particle and the NW is related to the flow of diffusing adatoms that become incorporated when they reach the circumference of the interface [4]. It yields a characteristic signature: the NW length varies like the inverse of the diameter. Such a behavior appears in Figure 5a for the case of the <111>-oriented Si NWs grown by MBE.

It is also visible after a 15 min for the growth of <110>-oriented Ge NWs (Figure 5b). Although the deposition rate was almost similar, comparison of the maximum length that can be reached between the <111> and <110>-oriented NWs clearly shows that the <110>-oriented NWs are quickly much longer. Based on the mass transport model described in [20], in the case of NWs that are still short, the length growth rate dependence on the adatoms diffusing onto the sidewalls from the substrate toward the interface between the Au droplet and the NW is expressed as follows:

(1) $$\frac{dL}{dt}=\frac{4\Omega \left|J\right|}{D\cosh\left(L/\lambda_{\text{f}}\right)}$$

where Ω, *J*, and *λ*_f _are, respectively, the atomic volume of the growth species, the flow of adatoms from the substrate toward the NW sidewalls, and the diffusion length along the NW sidewalls. Considering that Ω and *J *do not significantly vary between the growth of Si and Ge NWs, for a given diameter, the length growth rate is found to vary as the inverse of a cosh function that depends on *λ*_f_. A higher diffusion length on the sidewalls results in an increase of the length growth rate.

Surface diffusion requires overcoming an energy barrier [21]. The smaller the surface corrugation is, the lower the activation energy is. For a given time, elemental Si or Ge can thus diffuse further away from their adsorption site when the surface is atomically flat [22]. The longest diffusion length found for the case of <110>-oriented Ge NWs is, therefore, consistent with atomically flat {111} sidewalls in comparison with the rough-facetted {112} sidewalls of the <111>-oriented NWs.

**Figure 6:**
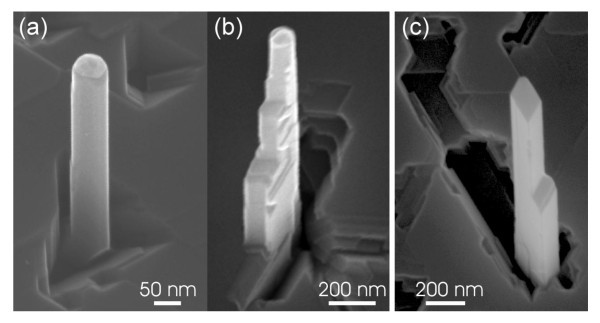
**Evolution of the <110>-oriented Ge NW morphology**. The NWs initially show **(a) **an irregular hexagonal cross-section, then **(b) **a reduction of the gold seed particle and a lateral overgrowth on the {111} sidewall that is exposed to the Ge flux, and finally **(c) **the disappearance of both the gold seed particle and the {100} sidewalls, giving rise to Ge NW with a rhombohedral cross-section.

When *L *becomes larger than *λ*_f_, then the length growth rate depends mainly on the diffusion of adatoms that adsorb directly onto the NW sidewalls or on the seed particle. Therefore, the NWs with the smallest diameters grew slower while the NWs with the biggest diameters keep on growing with the same length growth rate. The effect of a limited diffusion length is readily visible in Figure 6. While the <110>-oriented Ge NWs appear cylindrical at the beginning of the growth (Figure 6a), some of the {111} sidewalls may show a change of their morphology, as the growth proceeds. Such an example is seen in Figure 6b, where a strong overgrowth occurs at the base of the NW indicating that the adatoms from the substrate are rather incorporated onto the sidewalls than diffusing up to the top of the NWs. Finally, when the growth duration approaches tens of minutes, Au may have completely diffused away from the original seed particle. As a result, the NW cannot grow in length any more, but overgrowth on the sidewalls occurs. The {100} sidewalls, for which the surface tension is higher [15,23], disappear through the lateral growth of the {111} sidewalls, giving rise to a typical rhombohedral cross-section (Figure 6c).

In summary, by combining SEM and TEM analysis of group IV semiconductor NWs grown by MBE on a Si(111) surface, the structural properties of Si and Ge NWs with different growth axes have been investigated. As gold diffuses from the seed particle during the growth and wets the NW sidewalls, a significant change of the sidewall roughness can occur depending on the sidewall orientations. The roughness strongly affects the diffusion length of the diffusing Si or Ge adatoms toward the interface between the seed particle and the NW, and prevents the growth of NWs with micrometer-scale lengths. A good control of the NW growth axis is, therefore, important to obtain sidewalls with the lowest surface tension.

## Abbreviations

CVD: chemical vapor deposition; HRTEM: high-resolution transmission electron microscope; MBE: molecular beam epitaxy; NWs: nanowires; SEM: scanning electron microscope; UHV: ultra high vacuum.

## Competing interests

The authors declare that they have no competing interests.

## Authors' contributions

TX, JPN, BG designed the experiments, TX,JS,JPN performed the experiments, GP performed the TEM analyses, BG wrote the paper. All authors discussed the results and commented on the manuscript.
